# Marine Compounds and Cancer: 2017 Updates

**DOI:** 10.3390/md16020041

**Published:** 2018-01-24

**Authors:** Sergey A. Dyshlovoy, Friedemann Honecker

**Affiliations:** 1 Laboratory of Marine Natural Products Chemistry, G.B. Elyakov Pacific Institute of Bioorganic Chemistry, Far-East Branch, Russian Academy of Sciences, 690022 Vladivostok, Russia; 2 Laboratory of Experimental Oncology, Department of Oncology, Hematology and Bone Marrow Transplantation with Section Pneumology, Hubertus Wald-Tumorzentrum, University Medical Center Hamburg-Eppendorf, 20246 Hamburg, Germany; 3 School of Natural Sciences, Far East Federal University, 690022 Vladivostok, Russia; 4 Tumor and Breast Center ZeTuP St. Gallen, CH-9006 St. Gallen, Switzerland

By the end of 2017, there were seven marine-derived pharmaceutical substances that have been approved by the FDA for clinical use as drugs. Four of them are approved for the treatment of cancer, namely cytarabine (Cytosar-U^®^, first approved in 1969 for the treatment of leukemia), eribulin mesylate (Halaven^®^, first approved in 2010 for the treatment of metastatic breast cancer), brentuximab vedotin (Adcetris^®^, first approved in 2011 for the treatment of anaplastic large T-cell malignant lymphoma, and Hodgkin’s lymphoma), and trabectidine (Yondelis^®^, first approved in 2015 for the treatment of soft tissue sarcoma and ovarian cancer) [1]. Additionally, a number of marine-derived substances with potent anticancer properties are currently undergoing different stages of clinical development in oncology and hematology. Among them are plinabulin, plitidepsin, glembatumumab vedotin, and lurbinectedin (all in Phase III clinical trials); depatuxizumab mafodotin, AGS-16C3F, polatuzumab vedotin, PM184, tisotumab vedotin, and enfortumab vedotin (all in Phase II clinical trials); GSK2857916, ABBV-085, ABBV-399, ABBV-221, ASG-67E, ASG-15ME, bryostatin, marizomib, and SGN-LIV1A (all in Phase I clinical trials) [1]. Additionally, there are around 1500 natural molecules that were isolated from marine organisms, for which potent in vivo biological activity has been described, and more than 10,000 different compounds that have exhibited in vitro activity [2]. Therefore, natural compounds are a rich reservoir of molecules showing promising bioactivity, which will most certainly lead to the further development of potent anticancer compounds in the future.

To document this dynamic field of research, the topical collection “Marine Compounds and Cancer” (http://www.mdpi.com/journal/marinedrugs/special_issues/marine-compounds-cancer) of the open access journal *Marine Drugs* (ISSN 1660-3397) was started in 2017, one year after the special issue under the same name had been closed [3]. In 2017, a total of nine papers in this collection—two reviews and seven research articles—were published. The collection covers both novel and previously known anticancer agents from different classes of small and high molecular compounds, exploring their chemical structures and anticancer activity. Below, a short overview of the articles published in 2017 in the topical collection “Marine Compounds and Cancer” is provided.

A comprehensive review article by Ćetković and colleagues from Croatia reviews the current knowledge of **cancer-related genes/proteins found in marine sponges**. Elucidating cancer-associated genes in such simple and ancient animals as sponges may help to understand the more complex signaling interactions in higher animals [4]. Another review article by Martínez-Poveda and colleagues from Spain describes various biological activities of **puupehenones** (named after the famous rock Puu Pehe in Hawaii)—a large family of chemical compounds initially isolated from sponges of the orders Verongida and Dictyoceratida. This review gives an update on the current knowledge and understanding of the bioactivity and biogenesis of puupehenones, and their possible therapeutic applications in human diseases, with special emphasis on cancer [5].

Among the research articles, there is a report by Sarmiento-Vizcaíno and colleagues from Spain on **paulomycin G**—a novel molecule of the paulomycin family, isolated from a deep-sea sediment-derived micromonospora matsumotoense M-412. This compound demonstrates moderate cytotoxic activity in human cancer cells, but no antibacterial or antifungal activity [6]. Sperlich and colleagues from Germany and Canada show that the marine diterpene glycosides **pseudopterosins** have the ability to block the major inflammatory signaling pathway NF-κB by inhibiting the phosphorylation of p65 and IκB in leukemia and breast cancer cells, resulting in a reduction of the pro-inflammatory cytokines IL-6, TNFα, and MCP-1. They hypothesize that pseudopterosins inhibit NF-κB through activation of the glucocorticoid receptor in triple negative breast cancer [7]. Sun and colleagues from China report anticancer activity of a **selenium-containing polysaccharide-protein complex**, isolated from the selenium-enriched algae *Ulva fasciata*. Functionally, they describe the ability of this complex to induce mitochondria-mediated apoptosis in human lung cancer cells [8]. Xin and colleagues, representing another research group from China investigating marine substances, report the results of a virtual screening of low cytotoxic and non-cytotoxic natural products for their **ability to inhibit topoisomerase I**, which they consecutively confirmed experimentally by biological assays [9]. Another interesting research project performed by Dithmer and colleagues from Germany and the UK report unexpected cytoprotective activity of **fucoidan** in uveal melanoma cells as well as pro-angiogenic properties. Thus, despite the fact that fucoidan has previously been shown to also exhibit anti-tumorigenic effects, the authors conclude that this polysaccharide has no potential as a novel therapy for this type of cancer [10]. Hegazy and colleagues from Egypt, Japan and the USA report the isolation of three new cembrene diterpenoids—**sarcoehrenbergilid A–C**—along with four known diterpenoids and one steroid. The compounds were isolated from the Red Sea soft coral *Sarcophyton ehrenbergi*. The biological studies revealed moderate cytotoxic activity of these compounds in human cancer cell lines. Interestingly, additional molecular docking studies predict a high inhibitory activity of some of the compounds against the kinase domain of the growth receptor EGFR [11]. Schirmeister and colleagues from Germany and Italy describe the isolation of a new **6-epi-plakortide H acid** along with several previously known compounds from the caribbean sponge *Plakortis halichondrioides*. The compounds exhibit potent cytotoxic activity in both sensitive and multidrug-resistant human leukemia cells [12]. Finally, Xu and colleagues from China isolated two new **brevianamides** and two new **mycophenolic acid** derivatives, along with several previously known compounds, from the deep-sea-derived fungus *Penicillium brevicompactum* DFFSCS025. Among others, one compound exhibits moderate cytotoxicity against human colon cancer HCT116 cells [13].

In summary, our updated topical collection of *Marine Drugs*, “Marine Compounds and Cancer”, compiles recent results of research activities in the field of anticancer marine compounds from the year 2017. Exerting the honorable task of editing this collection, we express our gratitude to all authors who contributed. We are all looking forward to new and exciting discoveries!

Dr. Sergey A. Dyshlovoy and Dr. Friedemann Honecker,

Guest Editors of “Marine Compounds and Cancer”, and Editorial Board Members of *Marine Drugs*

## References

[1] Mayer A. Marine Pharmaceutical: The Clinical Pipeline. http://marinepharmacology.midwestern.edu/clinPipeline.htm.

[2] Mayer A. The marine pharmacology and pharmaceuticals pipeline in 2017. Proceedings of the 10th European Conference on Marine Products.

[3] Dyshlovoy S.A., Honecker F. (2015). Marine compounds and cancer: Where do we stand?. Mar. Drugs.

[4] Cetkovic H., Halasz M., Herak Bosnar M. (2018). Sponges: A reservoir of genes implicated in human cancer. Mar. Drugs.

[5] Martinez-Poveda B., Quesada A.R., Medina M.A. (2017). Pleiotropic role of puupehenones in biomedical research. Mar. Drugs.

[6] Sarmiento-Vizcaino A., Brana A.F., Perez-Victoria I., Pérez-Victoria I., Martín J., de Pedro N., Cruz M., Díaz C., Vicente F., Acuña J.L. (2017). Paulomycin G, a New Natural Product with Cytotoxic Activity against Tumor Cell Lines Produced by Deep-Sea Sediment Derived Micromonospora matsumotoense M-412 from the Aviles Canyon in the Cantabrian Sea. Mar. Drugs.

[7] Sperlich J., Kerr R., Teusch N. (2017). The marine natural product pseudopterosin blocks cytokine release of triple-negative breast cancer and monocytic leukemia cells by inhibiting NF-κB signaling. Mar. Drugs.

[8] Sun X., Zhong Y., Luo H., Yang Y. (2017). Selenium-containing polysaccharide-protein complex in se-enriched ulva fasciata induces mitochondria-mediated apoptosis in A549 human lung cancer cells. Mar. Drugs.

[9] Xin L.T., Liu L., Shao C.L., Yu R.-L., Chen F.-L., Yue S.-J., Wang M., Guo Z.-L., Fan Y.-C., Guan H.-S. (2017). Discovery of DNA Topoisomerase I Inhibitors with Low-Cytotoxicity Based on Virtual Screening from Natural Products. Mar. Drugs.

[10] Dithmer M., Kirsch A.M., Richert E., Fuchs S., Wang F., Schmidt H., Coupland S.E., Roider J., Klettner A. (2017). Fucoidan does not exert anti-tumorigenic effects on uveal melanoma cell lines. Mar. Drugs.

[11] Hegazy M.F., Elshamy A.I., Mohamed T.A., Hamed A.R., Ibrahim M.A.A., Ohta S., Paré P.W. (2017). Cembrene diterpenoids with ether linkages from sarcophyton ehrenbergi: An anti-proliferation and molecular-docking assessment. Mar. Drugs.

[12] Schirmeister T., Oli S., Wu H., della Sala G., Costantino V., Seo E.-J., Efferth T. (2017). Cytotoxicity of endoperoxides from the caribbean sponge plakortis halichondrioides towards sensitive and multidrug-resistant leukemia cells: Acids vs. esters activity evaluation. Mar. Drugs.

[13] Xu X., Zhang X., Nong X., Wang J., Qi S. (2017). Brevianamides and mycophenolic acid derivatives from the deep-sea-derived fungus penicillium brevicompactum DFFSCS025. Mar. Drugs.

